# Influence of N-Acetylcysteine Supplementation on Physical Performance and Laboratory Biomarkers in Adult Males: A Systematic Review of Controlled Trials

**DOI:** 10.3390/nu15112463

**Published:** 2023-05-25

**Authors:** Diego Fernández-Lázaro, Carlos Domínguez-Ortega, Natalia Busto, Mirian Santamaría-Peláez, Enrique Roche, Eduardo Gutiérez-Abejón, Juan Mielgo-Ayuso

**Affiliations:** 1 Department of Cellular Biology, Genetics, Histology and Pharmacology, Faculty of Health Sciences, University of Valladolid, Campus of Soria, 42004 Soria, Spain; 2 Neurobiology Research Group, Faculty of Medicine, University of Valladolid, 47005 Valladolid, Spain; 3 Research Group “Nutrition and Physical Activity”, Spanish Nutrition Society “SEÑ”, 28010 Madrid, Spain; 4 Hematology Service of “Santa Bárbara Hospital”, Castile and Leon Health (SACyL), 42003 Soria, Spain; 5 Hematology Service of “Latorre Hospital”, 42004 Soria, Spain; 6 Department of Health Sciences, Faculty of Health Sciences, University of Burgos, 09001 Burgos, Spain; 7 Department of Applied Biology-Nutrition, Institute of Bioengineering, University Miguel Hernandez, 03202 Elche, Spain; 8 Alicante Institute for Health and Biomedical Research (ISABIAL), 03010 Alicante, Spain; 9 CIBER Physiopathology of Obesity and Nutrition (CIBEROBN), Carlos III Health Institute (ISCIII), 28029 Madrid, Spain; 10 Pharmacological Big Data Laboratory, Faculty of Medicine, University of Valladolid, 47005 Valladolid, Spain; 11 Pharmacy Directorate, Castilla y León Health Council, 47007 Valladolid, Spain

**Keywords:** N-acetylcysteine, sport supplementation, physical performance, safety, oxidative stress, antioxidant, glutathione homeostasis, laboratory biomarkers

## Abstract

N-acetylcysteine (NAC) is used as a sports supplement for its ability to modulate exercise-induced oxidative damage through its antioxidant actions and maintenance of glutathione homeostasis, positioning NAC as a strategy to improve physical performance. We aimed to evaluate the current evidence on the benefits of NAC supplementation on physical performance and laboratory biomarkers in adult men. Using the Preferred Reporting Items for Systematic Reviews and Meta-Analyses (PRISMA) guidelines, we systematically reviewed studies indexed in the Web of Science, Scopus, and PubMed to assess the effects of NAC on physical performance, laboratory biomarkers, and adverse effects in adult men. Original articles published up to 30 April 2023 with a controlled trial design comparing NAC supplementation with a control group were included. The modified McMaster Critical Review Form for Quantitative Studies was used as an assessment tool and the Cochrane Risk of Bias was applied. Of the 777 records identified in the search, 16 studies met the inclusion and exclusion criteria. Overall, most of the trials reported beneficial effects of NAC supplementation and no serious adverse events were reported. Participants supplemented with NAC showed significant improvements in exercise performance, antioxidant capacity, and glutathione homeostasis. However, there was no clear evidence of beneficial effects of NAC supplementation on haematological markers, inflammatory response, and muscle behaviour. NAC supplementation appears to be safe and may regulate glutathione homeostasis, have antioxidant effects, and improve exercise performance. However, further studies are needed to clarify the relevance of its use.

## 1. Introduction

Oxidative stress (OS) is the imbalance between the physiological mechanisms responsible for the production and neutralisation of reactive compounds capable of causing oxidative molecular damage [1]. Jones et al. [2] defined OS as “the imbalance between oxidants and antioxidants in favour of the former, resulting in a breakdown of the physiological control and signalling normally exerted by the redox system, leading to molecular damage”. In this sense, molecular damage is the fundamental condition to speak of a true imbalance or OS. In addition, the oxidising and antioxidant compounds of the redox system play a very important role in the homeostasis of biological systems [3]. The induction of OS during physical exercise, especially during strenuous and high-intensity exercise, generates a greater amount of reactive oxygen species (ROS) that can cause OS in our bodies and produce adverse effects on the body [4]. This can lead to cell damage at the level of the myocyte membrane, or to an exacerbated inflammatory response, resulting in excessive pain, premature fatigue, and ultimately, injury [5].

In addition, there are extrinsic factors to exercise that can increase and trigger more OS in the body or impair the effectiveness of the antioxidant defence system, such as environmental conditions and the athlete’s diet [6]. These factors can be considered oxidative risk co-factors, as they increase the risk of damage and OS due to their cumulative effect on the ROS sources of exercise [7]. In these cases, the administration of exogenous antioxidants seems necessary to alleviate oxidative damage [8]. To this end, several antioxidants are currently on the market that can be administered as dietary supplements [9]. Supplementation with antioxidants in conjunction with physical activity would potentially reduce the harmful effects of exercise-induced OS, enhance the antioxidant defence system, and increase the beneficial effects of physical activity by improving exercise performance [10].

The potential antioxidant and anti-inflammatory effects of N-acetylcysteine (NAC) [2R]-2-acetamido-3-sulphanylpropanoic acid have been described since the 1960s, although in the last decade of the twentieth century, studies focused on its action as a mucolytic agent in the 1990s [11]. Subsequent studies have therefore been devoted to evaluating its use in the field of physical activity and determining its efficacy as a sports supplement to improve health and performance [11]. The World Anti-Doping Agency (WADA) does not include NAC in its list of banned substances [12]. NAC is a low molecular weight thiol containing the functional group formed by a sulphur atom and a hydrogen atom (-SH), where sulphur is the analogue of a hydroxyl group (-OH) [13]. The amino acid NAC can modulate OS through its actions as a cysteine donor in maintaining glutathione homeostasis and through a direct knockdown of ROS [14]. In addition, NAC reduces exercise-induced inflammation and fatigue through its thiol content, promoting the up-regulation of anti-inflammatory cytokines and minimising skeletal muscle damage after exhaustion of contractile activity [15]. However, Rhodes et al. [16] after conducting a systematic review, did not observe improvements in physical performance in ill, untrained, and trained participants using different dose ranges and posology. Overall, the aim of this study was to examine the current information available through a systematic review of the effects of NAC on exercise and laboratory biomarkers, and to assess whether NAC supplementation improves physical performance, antioxidant status, glutathione homeostasis, inflammatory response, immune function, haematological biomarkers, muscle behaviour, and side effects in healthy adult males, physically active healthy adult males, and athletic males without chronic disease.

## 2. Materials and Methods

### 2.1. Protocol and Registration

This review was conducted and reported according to the PRISMA (Preferred Reporting Items for Systematic Reviews and Meta-analyses) guidelines [17]. As this review was eligible for PROSPERO registration, it was registered for public access to avoid unnecessary duplication (#CRD42023418234).

### 2.2. Elegibility Criteria

The following inclusion criteria were applied for the selection of studies: (i) healthy, physically active healthy adults or athletes (different sports modalities); (ii) clinical trials (randomised or not); (iii) studies evaluating outcomes (primary or secondary) of exercise performance and laboratory biomarkers; (iv) studies clearly reporting the dose, frequency, and route of NAC administration; (v) languages were limited to English and Spanish; (vi) studies with a risk of bias score ≥ 4 according to the Cochrane Collaboration tool [18]; (vii) articles with a methodological quality score ≥ 13 according to the McMaster University Occupational Therapy Evidence-Based Practice Research Group for quantitative studies [19]. Registers that were not original research (editorials, notes, reviews, dissertations) or included adults (children, elderly) were excluded.

### 2.3. Information Sources

A structured search was carried out in electronic databases: Medline (PubMed), SCOPUS, and the Web of Science (WOS) between January 2023 and April 2023, published since the inception of the database, limited to English and Spanish language articles, and based on the PRISMA guidelines [17]. All high-quality databases guarantee good bibliographic support.

### 2.4. Search Methods

The search strategy included terms related to NAC and the different outcome labour and sport biomarkers, as well as a combination of these using the Medical Subject Headings (MeSH) index and Boolean operators: (“Acetylcysteine” OR “N-acetyl cysteine” OR “N-acetylcysteine” AND (“Athletes” OR “Sports” OR “Athletic Performance” OR “exercise/physiology” OR “muscle, skeletal” OR “Physical Fitness” OR “ Cardiorespiratory Fitness”) AND (“Adaptations” OR “Markers” OR “Effects” OR “Analysis” OR “Biomarkers” OR “Indicators” OR “Activity” OR “Pathways) NOT (“Syndrome” OR “Disease” or “Therapy” Or “Wounds and injuries”).

### 2.5. Methodological Quality Assessment

The methodological quality of the articles was assessed using the McMaster University Occupational Therapy Evidence-Based Practice Research Group [19], a tool designed to assess the methodological quality of clinical designs.

### 2.6. Risk of Bias Assessment

The Cochrane risk of bias tool was used [18]. This tool assesses the heterogeneity of the results of the selected trials. It consists of 8 items assessing selection bias (items 1 and 2), performance bias (item 3), detection bias (item 4), attrition bias (item 5), notification bias (item 6), publication bias (item 7), and observer bias (item 8).

### 2.7. Study Selection

The review was completely independently carried out: titles, abstracts, and full texts; by two reviewers (D.F.-L., J.M.-A.). In addition, the inclusion criteria were independently assessed, and disagreements were resolved by a second reviewer (C.D.-O.). No additional records of reference lists of relevant articles or grey literature were made. In addition, two study investigators constructed a network graph using the Connected Papers website (www.connectedpapers.com, accessed on 19 April 2023) to ensure inclusion of publications through visual characterisation of records.

### 2.8. Data Extraction

According to the CONSORT Statement for Control Trials 2010 [20], the following data were collected: name of the first author, year of publication, country in which the study was conducted, study design, sample size, sex and age of participants, duration of intervention, dose, and route of treatment. This was carried out by two study investigators (D.F.-L., J.M.-A.) and disagreements were resolved by the intervention of another study investigator (C.D.-O.).

### 2.9. Sumary Measures

The primary outcome was changes in physical performance variables (blood lactate, power, maximum oxygen volume [VO_2_max], oxygen consumption, time to exhaustion, fatigue index [FI], total work, economy cycling, rating of perceived exertion [RPE], respiratory exchange ratio [RER], heart rate [HR], high-intensity exercise [HITe]), laboratory biomarkers (antioxidant status: total antioxidant capacity [TAC], superoxide dismutase [SOD]; = manganese superoxide dismutase [MnSOD], malonyl dialdehyde [MDA], xanthine oxidase [XO], = thiobarbituric acid reactive substances [TRABS], catalase [CAT]; glutathione homeostasis: reduced glutathione [GSH], oxidised glutathione [GSSG], total glutathione [TGSH], glutathione reductase [GR], glutathione peroxidase [GPx], cysteine [CySH], total cysteine [TCyS], cysteine-glutathione disulphide [CySSG], cystine [CySS]; Inflammatory response: Interleukin 6 [IL-6], Interleukin 11 [IL-11], Tumour Necrosis Factor Alpha [TNF-α], Monocyte Chemoattractant Protein 1 [MCP-1], Nuclear Factor Kappa Beta [NF-kß]; Immune function: Natural Killer [NK]; Lymphocyte type [CD+]; Haematological biomarkers: Haemoglobin [Hb], Haematocrit [Hct]; Erythropoietin [EPO]; Red Blood Cells [RBC]; Muscle Behaviour: Creatine Kinase [CK], muscle pain, muscle soreness) and adverse effects following NAC supplementation. These parameters were included as outcomes because they are commonly investigated in health biomarker studies and sports science. Selected publications that met all the requirements proceeded to the next stage of data analysis and synthesis, supplemented by the review authors using the above criteria.

## 3. Results

### 3.1. Study Selection

**Figure 1:**
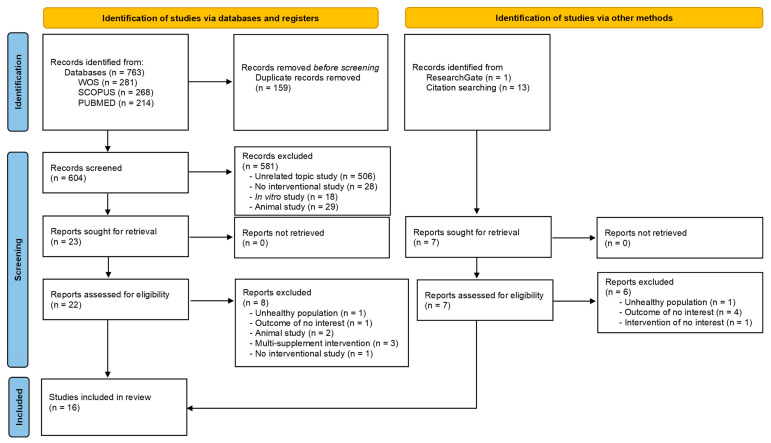
Flowchart of the methods used to search and select the literature.

The literature search yielded 777 studies, 763 from the electronic databases WOS, SCOPUS, and PubMed, and 14 from other sources such as ResearchGate and reference lists of relevant studies. After excluding 159 duplicates, a total of 604 articles identified in databases and registers were reviewed. After title and abstract evaluation, 23 articles were considered potential registries. After reviewing the full text and assessing potential records from databases, registries, and other sources, 16 [21,22,23,24,25,26,27,28,29,30,31,32,33,34,35,36] studies were included in the systematic review (Figure 1). 

**Figure 2:**
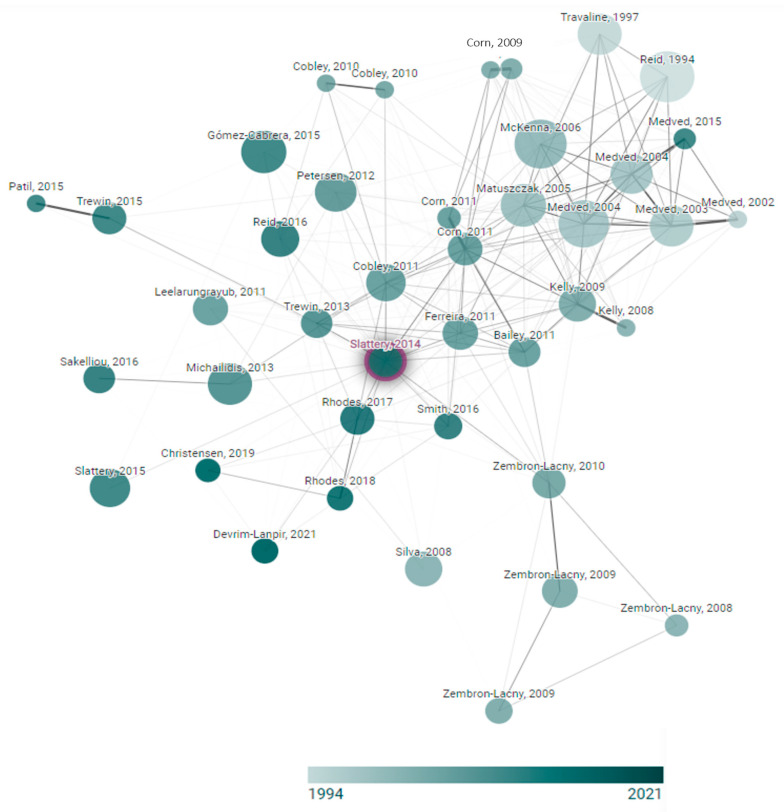
Network diagram of the N-acetylcysteine supplementation trials. This graph was developed within www.connectedpapers.com and accessed on 19 April 2023.

In addition, the verification of key records in the area of complementation with NAC is shown in Figure 2 through a graph that shows each node; we consider that the node graph originated from Slattery et al. [24].

### 3.2. Quality Assessment

Eight studies [21,22,23,24,25,29,33,36] were assessed as being “excellent” and eight [26,27,28,30,31,32,34,35] were assessed as being “very good”. All studies met the minimum quality criteria (Table 1).

### 3.3. Risk of Bias Assessment

Nine studies [21,22,23,25,29,31,32,35,36] had a score of “six points”, six studies [24,26,27,28,33,34] had a score of “five points”, and one study [30] had a score of “four points”. The main biases found in the studies included in the systematic review were items 1 and 4 (Table 2).

### 3.4. Characteristics of the Participants and Interventions

The number of total participants at baseline was 232 men. All of the participants were healthy individuals without any chronic conditions. Nine studies [21,24,25,27,28,31,33,35,36] included trained athletes: cycling [21], running or cycling [31,33,36], rowers [27,35], nonendurance trained [25], semi-elite rugby players [28], and triathletes [24], and seven studies included healthy [22,26,29,30,32] or healthy physically active individuals [23,34].

Intervention protocols varied by dose, duration, and schedule. Doses of NAC supplementation varied from 20 mg/kg [21] to 140 mg/kg [29], with 1200 mg/day as the most common oral dose used [25,26,30]. Furthermore, five studies [31,32,33,34,36] used two consecutive doses, 125 mg/kg/h during 15 min + 25 mg/kg/h, of intravenous solution. Supplementation duration ranged from 1 day [21,22,24,29,31,32,33,34,36] to 21 days [23]. Investigators administered supplementation 1 h before the test [21,22,24,29], before lunch plus dinner [25,26,27] or plus test [25], during the test [31,32,33,34,36], in the morning, 1–2 h prior to test [28], and during main meal [23,29,35]. Overall, subjects tolerated NAC supplementation well and no moderate or severe adverse reactions to NAC were observed during the supplementation, exercise, or post-supplementation periods (Table 3).

### 3.5. Outcome Evaluation

The data of the selected studies are summarized in Table 4.

### 3.6. Physical Performance

Six studies [22,25,26,31,32,33] included in the systematic review evaluated time to exhaustion; in three studies [22,31,33], a significant (*p* < 0.05) increase in the IG compared with the CG was observed. Furthermore, a significant (*p* < 0.05) improvement was also observed in the FI [30]. Two studies [22,24] reported significant (*p* < 0.05) improvements in power in the IG relative to the CG: Corn et al. [22] in peak power and Slattery et al. [24] in the average power evaluated at 5, 10, and 15 s of the test. Aerobic capacity was assessed by oxygen uptake [34] and VO_2_max [21,22,30,31], but only one study [31] described significant (*p* < 0.05) improvements in VO_2_max in the NAC-supplemented group compared to CG in highly endurance-trained participants. In four studies, anaerobic capacity was measured by blood lactate, only describing significant (*p* < 0.05) decreases in the IG compared to the CG in the study conducted by Leelarungrayub et al. [30]. Rhodes et al. [28] reported that NAC did not have an effect for a single bout of HITe (broken bronco shuttle test), but improved sprint performance of HITe in consecutive bouts (fastest shuttle test time).

### 3.7. Antioxidant Status

Of the three studies that evaluated TAC [21,24,30], two of these studies [24,30] showed that TAC significantly increased (*p* < 0.05) in participants supplemented with NAC compared to CG; in the same way, the ratio of pro-antioxidants significantly increased (*p* < 0.05) [27]. Three studies [24,26,27] evaluated TBARS levels, and all of them showed how TBARS concentration was significantly (*p* < 0.05) lower in the intervention group versus CG. In the studies included in the systematic review, no changes were reported in enzymatic activities such as CAT [27], SOD [27], and XO [24], and in MDA [23] metabolite in the IG compared to the non-supplemented group.

### 3.8. Glutathione Homeostasis

Six [22,25,26,29,32,33] clinical trials of the eight [22,24,25,26,29,32,33,34] studies that evaluated GSH showed significant (*p* < 0.05) increases in the IG versus CG. Furthermore, in endurance athletes (cyclists or runners), tGSH was significantly (*p* < 0.05) increased in muscle or plasma in the IG compared to the non-supplemented group [33]. In addition, GSSG levels were significantly reduced in two studies [29,33]; however, they significantly increased (*p* < 0.05) in [25] comparing both study groups (IG and CG). Furthermore, GPx activity was significantly (*p* < 0.05) increased in the IG compared to the CG [26,27]. However, no changes were observed in GR [27] activity in the IG compared to the non-supplemented group.

Five studies evaluated CySH and CySS levels [25,29,32,33,34]; in all of these studies, they observed significantly (*p* < 0.05) higher levels in the NAC-supplemented group compared to the non-supplemented group, and participants supplemented with NAC compared to CG, in the same way, the ratio of pro-antioxidants and protein thiols significantly increased (*p* < 0.05) [27]. Zembron-Lacny et al. [27] reported protein thiols significantly increased (*p* < 0.05) in the IG compared to the non-supplemented group.

### 3.9. Inflammatory Response

The inflammatory mediators IL-6 [24] and MCP-1 [24] significantly decreased (*p* < 0.05) in the IG compared to the CG. However, TNF-α were significantly (*p* < 0.05) increased when comparing both groups (CG and IG) [23]. The results of NF-κB were contradictory; Slattery et al. [24] described that NAC oral supplementation in triathletes significantly (*p* < 0.05) increased NF-κB, but intravenous administration of NAC in runners or cyclists significantly (*p* < 0.05) decreased NF-κB compared to the non-supplemented group [24]. Circulating levels of the anti-inflammatory cytokine IL-10 significantly (*p* < 0.05) increased in physically active healthy subjects in the IG compared to the CG [23].

### 3.10. Other Biomarkers

Nielsen et al. [35] evaluated the immune function and did not observe any changes between the IG and the CG.

Zembron-Lancy et al. [26], reported significant (*p* < 0.05) improvements in hematologic biomarkers such as Hb, Hct, EPO, RCB, MVC, and MHC in healthy students in the IG compared to the CG. However, McKenna et al. [31] did not observe changes in Hb and Hct when comparing both groups (GC and GC) in healthy subjects.

A significant (*p* < 0.05) reduction in muscle pain [23] was observed without changes in CK activity [30] in the IG compared to the CG. However, Rhodes et al. [28] increased muscle soreness.

### 3.11. Adverse Effects

Four studies [21,24,28,34] did not observe adverse reactions with NAC supplementation. Two studies [32,33] showed mild adverse reactions, such as erythema, vomiting, sweating, flushing, rashes, coughing, and itchy skin, and one study conducted by Ferreira et al. [29] noted mild to severe side effects such as gas, an upset stomach, nausea, and drowsiness. However, NAC supplementation did not induce immune alterations (NK or Lymphocytes) [35].

## 4. Discussion

This systematic review aimed to critically assess the effects of NAC supplementation on exercise performance and laboratory biomarkers in men: healthy adults, physically active healthy adults, and athletes without chronic medical conditions. Sixteen trials met the pre-specified inclusion/exclusion criteria. In general, participants supplemented with NAC showed significant improvements in exercise performance, antioxidant capacity, and glutathione homeostasis. However, there was no clear evidence of any beneficial effects of NAC supplementation on haematological markers, inflammatory response, and muscle behaviour.

### 4.1. N-Acetylcysteine Supplementation

NAC supplementation was administered by oral capsules [21,22,23,24,25,28,29,35], solution [29], or powder [26,27,30] and intravenous [31,32,33,34,36]. The doses of NAC used in interventions ranged from 9 mg/kg to 140 mg/kg [29], from 1 day [21,22,24,29,31,32,33,34,36] to 21 days [23], with no reports of serious adverse events. The adverse effects of NAC vary from mild to severe and depend on the pharmaceutical form and dose used, demonstrating that intravenous and oral NAC supplementation is associated with minimal side effects [15]. In addition, researchers have reported the occurrence of adverse effects such as flatulence, abdominal discomfort, nausea, pruritus, or erythema at doses higher than 20 mg/kg [29,32,33]. The number and severity of these side effects are proportional to the dose, with a maximum tolerated dose of 70 mg/kg [29] and a minimum effective dose of 9 mg/kg [29], since toxic effects in both adults and children only occur at doses of 6 g/kg when orally taken [37]. The Spanish Agency for Consumer Affairs, Food Safety, and Nutrition (AECOSAN) has proposed a maximum daily intake of 300 mg of NAC in food supplements [38]. NAC is not a doping agent [12].

### 4.2. Antioxidant Status

TAC is considered to be a reliable indicator of antioxidant content, which would measure the antioxidant capacity of the organism and therefore evaluate the efficacy of antioxidant supplements [39]. Two studies [24,30] showed that TAC significantly increased (*p* < 0.05) in IG, 9 days [24] and 7 days [30], compared to CG. However, the study conducted by Christensen et al. [21] reported no difference between the two groups (IG, CG) after 1 day of NAC supplementation. The direct antioxidant activity of NAC is due to the ability of its free thiol group to react with ROS, but the direct antioxidant activity of NAC is usually lower than that of other antioxidant supplements [15]. The concentration of NAC is considered to be a limiting factor in its direct antioxidant activity [40]. This may explain the differences in the results of the studies included in this systematic review [21,24,30]. Therefore, periods of continuous supplementation [24,27,30], of at least 3 days, would provide a higher concentration of NAC, and therefore a direct antioxidant effect, assessed by TAC [24,30] or pro-antioxidant ratio [27]. Ongoing supplementation with a multi-ingredient antioxidant supplement for 2 weeks [41] and 4 weeks [41] also increases TAC levels in athletes.

Exercise stimulates an increase in peroxidation that damages cell membranes, alters lipoproteins, and breaks down structures containing lipid conjugates [4]. Lipid peroxidation can be assessed using TBARS in biological samples [42]. Three studies [24,26,27] showed that TBARS levels were significantly (*p* < 0.05) lower in the NAC group than in the CG group. This is in line with Yalçin et al. [42] who reported that oral supplementation with 100 mg/day of NAC blocked lipid peroxidation in chronic blepharitis. The reduction in lipid peroxidation occurs as with other antioxidant supplements, such as vitamin C and/or E, and prevents OS [43,44].

### 4.3. Glutathione Homeostasis

NAC has antioxidant properties as a prodrug of intracellular CySH (intracellular increase in CySH concentration) with a subsequent increase in GSH. CySH is a building block and rate-limiting step of GSH [45]. CySS is another of the GSH precursor amino acids [46]. GSH is the most abundant non-protein thiol in the body and one of the major antioxidants against ROS, and GSH is a cofactor of GPx [40]. Thus, GSH [22,25,26,29,32,33], tGSH [33], and GPx [26,27] were significantly increased after NAC supplementation compared to CG. CySH and CySS [25,29,32,33,34] levels were also significantly (*p* < 0.05) increased in the NAC-supplemented group compared to the non-supplemented group. NAC appears to be a more appropriate supplement than GSH or CySH administration to modulate OS induced by drugs (paracetamol) or diseases that occur with low GSH levels, such as chronic obstructive pulmonary disease (COPD), by increasing intra-tissue GSH [47,48]. Other antioxidant supplements also increase tissue GSH levels through other pathways, such as curcumin activating the nuclear factor-like 2 (Nrf2) transcription factor pathway, which is integral to several antioxidant enzymes, including γ-glutamylcysteine synthase (an enzyme that catalyses the committed step in the synthesis of GSH) [8], selenium (Se), which participates in the expression of genes encoding GPx and GR (keys in the GSH redox cycle) [9], and vitamins C and E, which protect against oxidative degradation of GSH in the blood [49].

In addition, NAC is a precursor of free thiol proteins, which enhance GSH biosynthesis by degrading extracellular thiol proteins, such as cysteinylated proteins [15]. NAC also increases the concentration of protein thiols by converting GSSH to GSH [50]. There is a reservoir of low concentrations of GSSG in the endoplasmic reticulum, which acts as a source for the secretion of thiol proteins [51]. Thus, in a study of canoeists and rowers included in our systematic review, protein thiols were significantly increased (*p* < 0.05) in the IG compared to the CG [27].

### 4.4. Inflammatory Response

NAC has been linked to its anti-inflammatory activity, which favours the maintenance of cellular redox balance. NAC exerts a strong protective effect against inflammation in various conditions by reducing inflammatory mediators in animal models [52]. However, its efficacy in human clinical trials in various pathological conditions remains controversial [53]. For example, the effects of NAC supplementation on cytokine production were controversial in the trials included in this systematic review [23,24,30,35,36]. NAC supplementation showed anti-inflammatory activity by significantly (*p* < 0.05) reducing the inflammatory mediators IL-6 [24] and MCP-1 [24], and significantly(*p* < 0.05) increasing the anti-inflammatory cytokine IL-10 [23] in the IG compared to the CG. However, TNF-α was significantly (*p* < 0.05) increased when comparing both groups (CG and IG) [23]. TNF-α is the early-response, pro-inflammatory after strenuous exercise [54]. The increase in the TNF-α-supplemented group could be related to muscle damage altering a transition phase between intracellular TNF-α production and release [23].

It is possible that the mechanism by which NAC stimulates the production of anti-inflammatory cytokines after exercise is related to the decrease in NF-kB activation and expression of the c-Jun N-terminal kinase family (JNK) [15,53]. This premise was confirmed in the study by Petersen et al. [36] but Slattery et al. [24] reported a significant (*p* < 0.05) increase in NF-kB activation. High doses of NAC may be required for NF-kB downregulation, such as those intravenously administered [36], but oral supplemental doses by capsule [24] may be insufficient given the low bioavailability of NAC, between 6.45% and 10% [37]. NAC suppresses NF-κB activation at intracellular concentrations ≥ 10 mM [55].

The discrepancy in the inflammatory response could be due to related individual genetic differences in the response to different physical activity stimuli and in the production of mediators [56].

### 4.5. Other Biomarkers

The immunomodulatory properties of NAC at high doses (2400 mg/day) could elevate GSH levels in lymphocytes and modulate neutrophil functions during the development of SARS-CoV-2 infection, but NAC does not increase functions in the immune response [55]. Nielsen et al. [35] did not observe any changes in the immune response between IG and CG, because the immune function in the sailors was not affected by the response to OS.

The immunomodulatory properties of NAC at high doses (2400 mg/day) could increase GSH levels in lymphocytes and modulate neutrophil functions during the development of SARS-CoV-2 infection, but NAC does not enhance immune response [55]. Reported significant (*p* < 0.05) improvements in haematological biomarkers (Hb, Hct, EPO, RCB, MVC, and MHC) in the NAC group compared to the CG. According to Hildebrandt et al. [57] the redox state would regulate the function of O_2_ sensors involved in the response to hypoxia. NAC directly increases the concentration of thiols, and therefore, seems to modulate the EPO response, while at the same time attenuating OS. The increase in EPO secretion is mediated by an increase in hypoxia-inducible factor 1-alpha (HIF-1α), directly by NAC supplementation, or by plasma thiols that stabilise HIF-1α; for example, by acting on the hydroxylation of its proline residues [57]. Exercise-induced increases in the intraerythrocytic concentration of oxidised haemoglobin have been described [58]. NAC could act as a neutraliser of exercise-induced OS by protecting erythrocyte membranes [15], which would result in an increase in Hb and Hct values compared with non-supplemented subjects.

Myoglobin (Mb) (muscle haemoprotein) can auto-oxidise, generating superoxide anion and hydrogen peroxide, which induces exercise-induced muscle damage (EIMD) [59]. The increase in plasma thiol concentration induced by NAC acts as a stabiliser of the structural degradation of muscle proteins and provides an optimal/better state of skeletal muscle [14]. Thus, a significant (*p* < 0.05) reduction in muscle soreness was observed in the NAC supplementation group (10 mg/kg) compared to the placebo group in physically active healthy adults in the study by Silva et al. [23]. These results differ from the study by Martínez-Ferrán et al. [60]. These researchers reported that vitamin C plus vitamin E supplementation did not reduce soreness in endurance runners [60].

### 4.6. Physical Performance

The biological-metabolic mechanisms of NAC, through its antioxidant and anti-inflammatory actions, its maintenance of glutathione homeostasis, and effect on haematological, immunological, and biochemical biomarkers [15,45,52,57] as described in this systematic review, could make it an attractive sports supplement for potential use by athletes to improve athletic performance [16]. NAC was effective in improving aerobic performance [31], anaerobic performance [28,30], power output [22,24], time to exhaustion [22,31,33], overall exercise performance [33] fatigue [30], and muscle soreness [23]. This could suggest that NAC was effective when exercise was performed, and that NAC supplementation would have a sports-ergogenic effect. However, not all studies [21,22,24,25,26,28,30,32,34] have demonstrated performance benefits with NAC supplementation compared to CG. Therefore, the type and duration of supplementation, or the modality and duration of exercise, could influence the effect of NAC on the response and adaptations to exercise, making it difficult to make a clear judgement on the ergogenic potential of NAC as a sports supplement.

### 4.7. Limitations

The authors of this review acknowledge several limitations. Firstly, only a limited number of manuscripts met the inclusion criteria. Nevertheless, our systematic approach followed the PRISMA method [17], the search was conducted using three databases, PubMed, SCOPUS, and WOS, and included grey literature. In addition, we used the modified McMaster [19] methodological quality assessment tool and the Cochrane [18] risk of bias assessment tool to ensure that all selected records met the minimum quality criteria and included several outcomes commonly used in sports nutrition research. Secondly, there is a large heterogeneity of the studies in terms of outcomes, supplementation dose, and duration of intervention, which does not allow us to perform a meta-analysis. The large variability in NAC supplementation warrants caution in interpreting the results; however, there may be evidence of a benefit of NAC for adult men on exercise performance in healthy subjects, physically active healthy participants, and athletes.

### 4.8. Future Applications

This research could be of interest to sports physicians, nutritionists, and trainers who want to improve exercise performance, antioxidant capacity, and post-exercise glutathione homeostasis in their athletes. Taking advantage of the fact that most athletes simultaneously ingest different supplements at different times and doses. Multi-ingredient formulations could provide a better supplementation strategy that enhances athletic performance through the enhancement of laboratory biomarkers [59]. In this sense, Kumar et al. [61] recently described that the effects of NAC supplementation can be enhanced by adding glycine (Gly). NAC plus Gly supplementation improves glutathione deficiency, oxidative stress, mitochondrial dysfunction, inflammation, and physical function [61]. Perhaps NAC plus Gly would restore GSH deficiency, thus decreasing OS and mitochondrial abnormalities more than monotherapy (NAC or Gly) [62]. In addition, Cys is an important donor of methyl groups; in this sense [61], DNA methylation has been revealed as a fundamental epigenetic mechanism in the regulation of the expression of genes that control functions of muscle satellite cells crucial in the restoration of muscular damage [63].

## 5. Conclusions

The antioxidant, glutathione homeostasis, anti-inflammatory, haematological, and regulatory mechanisms make NAC the right supplement for athletes seeking to improve their athletic performance, but more evidence is needed to confirm these findings. The results reported in this systematic review also showed that NAC supplementation is safe. In general, more research on NAC supplementation and exercise performance is needed before clear conclusions can be drawn. Future studies should adequately investigate their supplementation and exercise methodology, as well as their outcomes. Further research should focus on the use of NAC supplementation in high-level or elite sports and attempt to adequately monitor and report adverse effects.

## Figures and Tables

**Table 1:** Results of the methodological quality assessment of included studies—McMaster Critical Review Form for Quantitative Studies [19].

Study, Year	Item																Total	%	Quality Score
	1	2	3	4	5	6	7	8	9	10	11	12	13	14	15	16			
Christensen et al. [21], 2019	1	1	1	1	1	1	1	1	1	1	1	1	1	1	1	0	15	93.8	E
Corn et al. [22], 2011	1	1	1	1	1	1	1	1	1	1	1	1	1	1	1	0	15	93.8	E
Ferreira et al. [29], 2011	1	1	1	1	1	1	1	1	1	1	1	1	1	1	1	1	16	100	E
Leelarungrayub et al. [30], 2011	1	1	1	0	1	0	1	1	1	1	1	1	1	1	1	0	13	81.3	VG
McKenna et al. [31], 2006	1	1	1	1	1	1	1	1	1	1	1	1	1	1	0	0	14	87.5	VG
Medved et al. [32], 2003	1	1	1	1	1	1	1	1	1	1	1	1	1	1	0	0	14	87.5	VG
Medved et al. [33], 2004	1	1	1	1	1	1	1	1	1	1	1	1	1	1	1	0	15	93.8	E
Merry et al. [34], 2010	1	1	1	1	1	0	1	1	1	1	1	1	1	1	1	0	14	87.5	VG
Nielsen et al. [35], 1998	1	1	1	1	1	0	1	1	1	1	1	1	1	1	1	0	14	87.5	VG
Petersen et al. [36], 2012	1	1	1	1	1	1	1	1	1	1	1	1	1	1	1	0	15	93.8	E
Rhodes et al. [28], 2019	1	1	1	0	1	1	1	1	1	1	1	1	1	1	0	0	13	81.3	VG
Silva et al. [23], 2008	1	1	1	1	1	1	1	1	1	1	1	1	1	1	1	0	15	93.8	E
Slattery et al. [24], 2014	1	1	1	1	1	1	1	1	1	1	1	1	1	1	1	0	15	93.8	E
Smith et al. [25], 2016	1	1	1	1	1	1	1	1	1	1	1	1	1	1	1	1	16	100	E
Zembron-Lancy et al. [27], 2007	1	1	1	0	1	1	1	1	1	1	1	1	1	1	0	0	13	81.3	VG
Zembron-Lancy et al. [26], 2010	1	1	1	0	1	1	1	1	1	1	1	1	1	1	0	0	13	81.3	VG

Abbreviations: 0 = not fulfilled criterion; 1 = fulfilled criterion; E = excellent; VG = very good; Item 1: study purpose; item 2: literature review; item 3: study design; item: 4 blinding; item 5: sample description; item 6: sample size; item 7: ethics and consent; item 8: validity of outcomes; item 9: reliability of outcomes; item 10: intervention description; item 11: statistical significance; item 12: statistical analysis; item 13: clinical importance; item 14: conclusions; item 15: clinical implications; item 16: study limitations.

**Table 2:** Results of the risk of bias assessment of included studies—Cochrane Bias Methods Group [18].

Study, Year	Items								
	1	2	3	4	5	6	7	8	Total
Christensen et al. [21], 2019	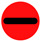	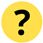	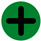	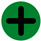	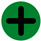	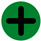	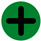	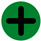	6
Corn et al. [22], 2011	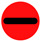	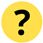	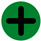	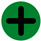	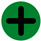	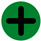	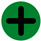	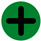	6
Ferreira et al. [29], 2011	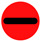	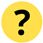	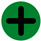	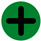	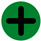	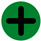	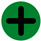	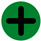	6
Leelarungrayub et al. [30], 2011	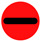	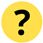	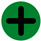	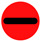	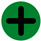	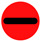	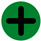	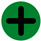	4
McKenna et al. [31], 2006	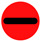	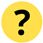	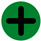	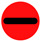	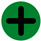	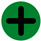	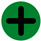	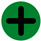	6
Medved et al. [32], 2003	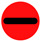	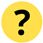	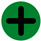	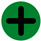	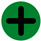	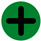	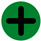	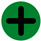	6
Medved et al. [33], 2004	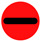	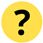	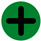	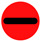	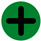	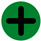	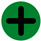	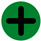	5
Merry et al. [34], 2010	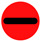	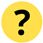	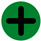	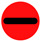	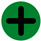	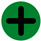	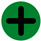	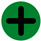	5
Nielsen et al. [35], 1998	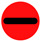	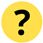	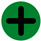	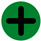	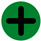	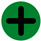	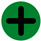	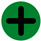	6
Petersen et al. [36], 2012	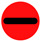	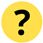	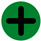	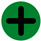	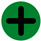	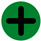	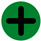	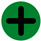	6
Rhodes et al. [28], 2019	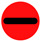	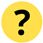	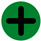	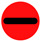	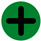	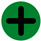	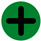	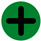	5
Silva et al. [23], 2008	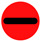	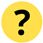	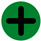	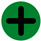	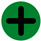	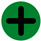	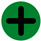	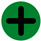	6
Slattery et al. [24]	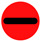	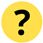	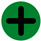	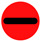	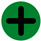	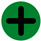	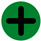	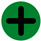	5
Smith et al. [25], 2016	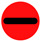	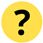	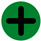	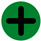	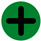	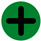	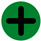	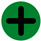	6
Zembron-Lancy et al. [27]	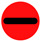	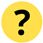	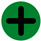	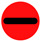	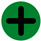	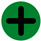	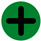	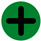	5
Zembron-Lancy et al. [26]	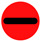	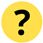	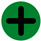	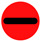	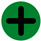	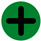	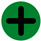	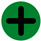	5

Abbreviations = 1: generation of sequences; 2: allocation concealment; 3: personal blinding; 4: blinding of assessor; 5: incomplete follow-up; 6: data report; 7: publication bias; 8: observer bias; the rating for each item includes the answer to one question, where “+” indicates bias, “−” indicates high risk of bias, and “?” indicates lack of information or uncertainty about the potential for bias; the higher the score, the greater the risk of bias.

**Table 3:** Characteristics of participants and supplementation protocols of the selected studies.

Characteristics	Types	Study
Participants	Healthy	[22,26,29,30,32]
	Healthy physically active	[23,34]
	Trained	[21,24,25,27,28,31,33,35,36]
Supplementation product	Registered product^®^	[21,22,24,27,28,29,30,34,35]
	No reported	[23,25,26,31,32,33,36]
Pharmaceutical form	Capsules	[21,22,23,24,25,28,29,35]
	Oral solution	[29]
	Powder	[26,27,30]
	Intravenous solution	[31,32,33,34,36]
Total dose	20 mg/kg	[21]
	72.3 mg/kg	[22]
	1000 mg/day (2 doses → 500 mg)	[28]
	1200 mg/day (2 doses → 600 mg)	[24,26,30]
	125 mg/kg/h during 15 min + 25 mg/kg/h during the test	[31,32,33,34,36]
	6000 mg/day	[35]
	10 mg/kg	[23]
	70 mg/kg	[25]
	1800 mg/day	[27]
	9, 18, 37, 70, 140 mg/kg	[29]
Duration (days)	1	[21,22,25,29,31,32,33,34,36]
	3	[27,35]
	6	[28]
	7	[30]
	8	[26]
	9	[24]
	21	[23]
Dose schedule	60 min prior test	[21,22,25,29]
	Morning, 1–2 h prior test	[28]
	Before lunch + before dinner	[24,26,27]
	During test	[31,32,33,34,36]
	Main Meal	[23,29,35]
	Before lunch + before dinner + prior test	[24]

Abbreviations: mg = milligrams; kg = kilograms; h = hour.

**Table 4:** Studies included in the systematic review of the effect of N-Acetylcysteine supplementation on health biomarkers.

First Author, Year of Publication, and Country	Study Design	Participants (Baseline Sample Size, Age, Sex, Withdrawals, and Final Group Sample Size)	Intervention	Outcomes	Results IG vs. CG
Christensen et al., 2019, Denmark [21]	Randomized, double-blind crossover, placebo-controlled trial	11 ♂ Well-trained cyclists Age (mean ± SD) 28 ± 7 years Height (mean ± SD) 183 ± 7 cm Body mass (mean ± SD) 73 ± 10 kg Peak VO_2_max (mean ± SD) 69 ± 7 mL/min/kg Study withdrawals: 1 (to illness) *n* = 10	1500 mg (20 mg/kg) capsules NAC (Fagron BV, Rotterdam, The Netherlands) 60 min before the test 1 day Washout period: 6 days	Peak Power Blood Lactate Cycling Economy VO_2_max TAC Adverse reactions	↔ Peak Power ↔ Blood Lactate ↔ Cycling Economy ↔ VO_2_max ↔TAC ↔ Side Effects
Corn et al., 2011 USA [22]	Randomized, double-blind crossover, placebo-controlled trial	7 ♂ Healthy Age (Range) 20–24 years Body mass (mean ± SD) 89.1 ± 11 kg Height (mean ± SD) 183 ± 5 cm Study withdrawals: 0	72.3 ± 1.3 mg/kg Capsules NAC (Physiologics, Northglenn, CO, USA) 60 min before the test 1 day Washout period: 3 days	Peak Power Time to exhaustion (80, 90, 100, 110%) VO_2_max GSH	↑* Peak Power ↑* Time to exhaustion 80% ↔ VO_2_max ↑* GSH
Ferreira et al., 2011 USA [29]	Randomized, double-blind crossover, placebo-controlled trial	17 ♂ Healthy Age (mean ± SD) 30 ± 2 years Body Weight (mean ± SD) 86 ± 5 kg	9 or 18 mg/kg capsules NAC (Physiologics, Northglenn, CO, USA), morning and evening before the test day 35.70 or 140 mg/kg liquid solution NAC (American Regent Laboratories Inc. Shirley, NY, USA), 60 min before the test 1 day Washout period: 7 days	GSH GSSG CySH CySS CySSG TGSH TCyS	↑* GSH (140 mg/kg) ↓* GSSG (70, 140 mg/kg) ↑* CySH ↑* CySS ↓* CySSG (70, 140 mg/kg) ↔ TGSH ↑* TCyS ↔ CySH:TCyS ratio (capsules) ↑* CySH:TCyS ratio (líquid)
Leelarungrayub et al., 2011 Thailand [30]	Randomized controlled trial	36 ♂ Healthy Age (range) 20–24 years Body mass index (range) 18.5–24.9 kg/m^2^ Study withdrawals: 7 16 participants IG 13 participants CG	1200 mg/day Two doses (600 mg) NAC effervescent powder (FLUIMUCIL A 600, ZAMBON Switzerland, Ltd., Cadempino, Switzerland) 7 days	Blood Lactate VO_2_máx % FI TAC TNF-α. CK	↓* Blood Lactate ↔ VO_2_máx ↑* % FI ↑* TAC ↔ TNF- α. ↔ CK
McKenna et al., 2006, Australia [31]	Randomized, double-blind crossover, placebo-controlled trial	7 ♂ High endurance trained (running or cycling) 4–5 times per week 1–2 h per day experience ≥ 2 years Age (mean ± SD) 27.1 ± 5.6 years, Weight (mean ± SD) 76.7 ± 10.9 kg Height (mean ± SD) 180 ± 5.4 cm Study withdrawals: 0	Intravenous infusion NAC 125 mg/kg/h for 15 min before test plus 25 mg/kg/h until the end of the test. Washout period: 7 days	Time to exhaustion VO_2_máx Hb Hct Na+/K+ pump activity Plasma K+ Plasma Electrolyte (Na^+^, Cl^−^, Ca^2+^) Acid–Base Status (HCO3^−^, PCO2, H^+^)	↑* Time to exhaustion ↑* VO_2_máx ↔ Hb ↔ Hct ↓* Na+/K+ pump activity ↓* Plasma K+ ↔ Plasma Electrolyte ↔ Acid–Base Status
Medved et al., 2003, Australia [32]	Randomized, double-blind crossover, placebo-controlled trial counterbalanced	8 ♂ Healthy Age (mean ± SD) 22.5 ± 2.4 years Body mass (mean ± SD) 77.81 ± 10.3 kg Height (mean ± SD) 177.6 ± 1.6 cm Study withdrawals: 0	Intravenous infusion NAC 125 mg/kg/h for 15 min before test plus 25 mg/kg/h until the end of the test. Washout period: 5–7 days	Time to exhaustion Total work (kJ) GSH GSSG CySH, CySS TGSH GSH:TGSH ratio Hb Hct Plasma Electrolyte (Na^+^, Cl^−^, Ca^2+^) K^+^ Acid–Base Status (HCO^3-^, PCO^2−^) Adverse reactions	↔ Time to exhaustion ↔ Total work (kJ) ↑* GSH ↓* GSSG ↑* CySH, ↑* CySS ↔ TGSH ↔ GSH:TGSH ratio ↔ Hb ↔ Hct ↔ Plasma Electrolyte (Na^+^, Cl^−^, Ca^2+^) ↑* K^+^ ↔ Acid–Base Status (HCO^3−^, PCO^2−^) ↑ Side Effects
Medved et al., 2004, Australia [33]	Randomized, double-blind crossover, placebo-controlled trial	8 ♂ Endurance trained (running or cycling) 4–5 times per week 1–2 h per day experience ≥ 2 years Age (mean ± SD) 27.1 ± 5.6 years Body mass (mean ± SD) 76.7 ± 10.9 kg Height (mean ± SD) 180.3 ± 5.4 cm Study withdrawals: 0	Intravenous infusion NAC 125 mg/kg/h for 15 min before test plus 25 mg/kg/h until the end of the test. Washout period: 5–7 days	Time to exhaustion Total work (kJ) GSH GSSG CySH, CySS TGSH GSH:TGSH ratio Adverse reactions	↑* Time to exhaustion ↑* Total work (kJ) ↑* GSH ↔ GSSG ↑* TGSH ↑* CySH (muscle; plasma) ↑* CySS (muscle; plasma) ↔ GSSG:TGSH ratio ↔ TSGH:GSH ratio ↑ Side Effects
Merry et al. 2010, Australia [34]	Randomized, double-blind crossover, placebo-controlled trial counterbalanced	9 ♂ Healthy physically active Age (mean ± SD) 23 ± 2 years Weight (mean ± SD) 79.7 ± 3.4 kg Height (mean ± SD) 179 ± 3 cm Study withdrawals: 0	Intravenous infusion NAC (Parvolex, Faulding Pharmaceuticals) 125 mg/kg/h for 15 min before test plus 25 mg/kg/h until the end of the test. Washout period: 14 days	Blood Lactate O_2_ Consumption HR RER RPE GSH GSSG GSH:GSSG ratio CySH (muscle; plasma) CySS S-glutathionylation Tyrosine nitration PCr, Cr ATP, ADP, AMP AMP:ATP ratio muscle glycogen Insulin NEFA Adverse reactions	↔ Blood Lactate ↔ O_2_ Uptake ↔ HR ↔ RER ↔ RPE ↔ GSH ↔ GSSG ↔ GSH:GSSG ratio ↑* CySH (muscle; plasma) ↑* CySS ↓* S-glutathionylation ↔ Tyrosine nitration ↔ PCr, Cr ↔ ATP, ADP, AMP ↔ AMP:ATP ratio ↔ muscle glycogen ↔ Insulin ↔ NEFA ↔ Side Effects
Nielsen et al., 1998, Denmark [35]	Randomized, double-blind crossover, placebo-controlled	14 ♂ Healthy oarsmen trained Age (mean ± SE) 27 ± 1 years Weight (mean ± SE) 80 ± 2 kg Height (mean ± SE) 189 ± 2 cm VO_2_max (mean ± SE) 5.1 ± 0.2 L/min	6000 mg/day 2 daily capsules NAC (ASTRA, Copenhagen, Denmark), 3000 mg Morning and evening meals for 3 days before the experiment, and 2 h before the exercise protocol Washout period: 21 days	Lymphocytes CD3+ CD4+ CD8+ CD14+ CD16+ CD19+ CD56+ NK activity	↔ Lymphocytes ↔ CD3+ ↔ CD4+ ↔ CD8+ ↔ CD14+ ↔ CD16+ ↔ CD19+ ↔ CD56+ ↔ NK activity
Petersen et al., 2012, Australia [36]	Randomized, double-blind crossover, placebo-controlled	8 ♂ Endurance trained (running or cycling) 4–5 times per week 1–2 h per day experience ≥ 2 years Age (mean ± SD) 27.1 ± 5.6 years Body mass (mean ± SD) 76.7 ± 10.9 kg Height (mean ± SD) 180.3 ± 5.4 cm VO_2_peak 65.6 ± 2.2 mL/kg Study withdrawals: 0	Intravenous infusion NAC 125 mg/kg/h for 15 min before test plus 25 mg/kg/h until the end of the test. Washout period: 7 days	MnSOD JNK ERK1/2 p38 MAPK NF-kB/p65 IkBα IL-6 MCP-1 HSP70 PGC-1α	↓* MnSOD ↓* JNK ↔ ERK1/2 ↔ p38 MAPK ↓*NF-kB/p65 ↔ IkBα ↔ IL-6 ↔ MCP-1 ↔ HSP70 ↔ PGC-1α
Rhodes et al, 2019, Australia [16]	Double-blind, pre-post, placebo-controlled	17 ♂ Semi-professional/Semi-elite rugby players Age (mean ± SD) 20.4 ± 0.9 years Weight (mean ± SD) 103.0 ± 12.0 kg Height (mean ± SD) 182.3 ± 7.4 cm Yo-Yo Intermittent Recovery Test Level 1 (mean ± SD) 17.4 ± 1.73 Level Study withdrawals: 4 6 participants IG 7 participants CG	IG: 1 g/day (2 × 50 mg capsules) NAC (Nutrabio Labs Inc., Middlesex, NJ, USA) CG: 1 g (2 × 50 mg capsules) of placebo (sucrose and salt mixture) For 6 days.	Muscle soreness Broken bronco shuttle test Fastest shuttle time (High-intensity exercise) Side effects	↑ Muscle Soreness ↔ Broken bronco shuttle test ↑ Fastest shuttle time ↔ Side Effects
Silva et al. 2008, Brazil [23]	Randomized, controlled, single-blind trial	29 ♂ Healthy physically active Age (mean ± SD) 21.3 ± 4 years Weight (mean ± SD) 74.5 ± 7.7 kg Height (mean ± SD) 177.2 ± 6.9 cm Study withdrawals: 4 8 participants IG 9 participants CG 8 participants IG + CG	1 capsule/day 10 mg/kg NAC 14 days before the eccentric exercise protocol and 7 days after exercise CG (21 days; placebo) IG (21 days; NAC) IG + CG (14 days NAC + 7 days placebo)	MDA Carbonylation levels TNF-α IL-10 Muscle pain	↔ MDA ↔ Carbonylation levels ↑* TNF- α ↑*IL-10 ↓ Muscle Pain
Slattery et al., 2014, Australia [24]	Randomized, double-blind crossover, placebo-controlled	10 ♂ Well-trained triathletes Age (mean ± SD) 23.6 ± 3.2 years Weight (mean ± SD) 70.5 ± 7.2 kg Height (mean ± SD) 179.8 ± 4.4 cm VO_2_max (mean ± SD) 663.3 ± 4.8 mL/kg/min Study withdrawals: 2 (injury and illness)	1200 mg NAC (Batch: 254709, The Melbourne Food Ingredient Depot, Victoria, Melburne, Australia), 2 * 600 mg capsules 9 days and 2 h before the test Washout period: 21 days	Average Power 5, 10, 15 s Total work Blood lactate RPE TAC GSH GSSG GSH: GSSG ratio XO TRABS FRAP IL-6, MPC-1 NF-kB Adverse reactions	↑* Average Power 5, 10, 15 s ↔ Total work ↔ Blood lactate ↔ RPE ↑* TAC ↔ GSH ↔ GSSG ↔ GSH: GSSG ratio ↔ XO ↓* TRABS ↔ FRAP ↓* IL-6 ↓* MPC-1 ↑* NF-kB ↔ Adverse reactions
Smith et al., 2016, USA [25]	Randomized, double-blind crossover, placebo-controlled	10 ♂ Non-endurance trained Age (mean ± SD) 21.8 ± 1.2 years Weight (mean ± SD) 77.1 ± 17.5 kg Height (mean ± SD) 174.9 ± 9.3 cm Peak Power (mean ± SD) 6.0 ± 1.3 W Study withdrawals: 0	70 mg/kg NAC 600 mg/capsule 60 min before the test Washout period: 7 days	Time to Exhaustion GSH GSSG GSH: GSSG CySH CySS BABF DAB	↔ Time to Exhaustion ↑* GSH ↑* GSSG ↓* GSH: GSSG ↑* CySH ↑* CySS ↔ BABF ↔ DAB
Zembron-Lancy et al., 2007, Poland [27]	Randomized placebo-controlled	30 ♂ Healthy young trained (Canoeists and Rowers) Age (mean ± SD) CG 21.5 ± 1.4 years IG 21.9 ± 1.7 years Body Mass (mean ± SD) CG 87.2 ± 10.6 kg IG 87.1 ± 12.8 kg Height (mean ± SD) CG 181.7 ± 8.3 cm IG 180.7 ± 7.4 cm Body Fat (mean ± SD) CG 14.4 ± 4.6 % IG 14.5 ± 5.6 % Study withdrawals: 0 15 participants IG 15 participants CG	IG: 1800 mg/day NAC (Hexal AG, Holzkirchen, Germany) as powder dissolved in 50 mL water CG: 3 × 350 mg/day *Saccharum Lactis* as powder dissolved in 50 mL water	Protein Thiols SOD GPx CAT TBARS Pro-Antioxidant ratio	↑* Protein Thiols ↔ SOD ↑* GPx ↔ GR ↔ CAT ↓* TBARS ↑* Pro-Antioxidant ratio
Zembron-Lancy et al., 2010, Poland [26]		15 ♂ Healthy students Age (mean ± SD) 20.3 ± 2.3 years Body Mass (mean ± SD) 83.4 ± 14.4 kg Height (mean ± SD) 180.0 ± 1.0 cm Study withdrawals: 0 8 participants IG 7 participants CG	IG: 1200 mg/day NAC 2 daily doses (1st dose in the morning in a fasted state and the second dose 2 h before an evening meal) for 8 days prior to and 1 dose 600 mg on the day of exercise trial Each dose as powder dissolved in 50 mL of water CG: Lactose as powder dissolved in 50 mL of water	Time to exhaustion Peak Power GSH GPx GR PC TBARS EPO Hb Hct MVC MHC RCB	↔ Time to exhaustion ↔ Peak Power ↑* GSH ↑* GPx ↑* GR ↓* PC ↓* TBARS ↑* EPO ↑* Hb ↑* Hct ↑* MVC ↑* MHC ↓* RCB

Abbreviations: ↑ = no significant increase; ↓ = no significant decrease; ↔ = no significant change. ↑* = significant increase; ↓* = significant decrease; *: Indicates significant values (*p* < 0.05); CG: control Group; IG: Intervention Group; NAC = N-acetylcysteine; SD = Standard deviation; VO_2_max = Maximum Oxygen Volume; O_2_ = Oxygen; FI = Fatigue Index; HR = Hear Rate; RPE = Rating of Perceived Exertion; RER = Respiratory Exchange Ratio; TAC = Capacity Antioxidant Total; GSH = Reduced Glutathione; GSSG = Oxidated Glutathione; TGSH = total glutathione; CySH = Cysteine; TCyS = Total Cysteine; CySSG = cysteine glutathione disulphide; CySS = Cystine; SOD = Superoxide Dismutase; MnSOD = Manganese Superoxide Dismutase; MDA = Malonyl Dialdehyde; XO = Xanthine Oxidase; TRABS = Thiobarbituric Acid Reactive Substances; FRAP = iron reducing capacity; CAT = Catalase; GR = Glutathione Reductase; GPx = Glutathione Peroxidase; CK = Creatine Kinase; TNF-α = Tumour Necrosis Factor Alpha; Hb = Haemoglobin; Hct = Haematocrit; PCr = Phosphocreatine; Cr = Creatine; ATP = Adenosine triphosphate; ADP = Adenosine Diphosphate; AMP = Adenosine Monophosphate; NEFA = Non-Esterified Fatty Acids; NK = Natural Killer; JNK = C-Jun-terminal Kinase; MCP-1 = monocyte chemoattractant protein 1; MAPK = mitogenic activation protein kinase; NF-kB = nuclear factor kappa B; IκBα = nuclear factor of kappa light polypeptide gene enhancer in B-cells inhibitor alpha; IL = Interleukin; PGC-1a = peroxisome proliferator-activated receptor coactivator 1α; HSP-70 = Heat Shock Proteins 70; PC = Carbonyl Proteins.

## Data Availability

All the data are in the manuscript.
